# Protection of Cells against Oxidative Stress by Nanomolar Levels of Hydroxyflavones Indicates a New Type of Intracellular Antioxidant Mechanism

**DOI:** 10.1371/journal.pone.0060796

**Published:** 2013-04-18

**Authors:** Emanuele Lombardo, Cristian Sabellico, Jan Hájek, Veronika Staňková, Tomáš Filipský, Valentina Balducci, Paolo De Vito, Stefano Leone, Eugenia I. Bavavea, Ilaria Proietti Silvestri, Giuliana Righi, Paolo Luly, Luciano Saso, Paolo Bovicelli, Jens Z. Pedersen, Sandra Incerpi

**Affiliations:** 1 Department of Sciences, University Roma Tre, Rome, Italy; 2 Department of Biochemical Sciences, Faculty of Pharmacy in Hradec Králové, Charles University, Prague, Czech Republic; 3 Department of Pharmacology and Toxicology, Faculty of Pharmacy in Hradec Králové, Charles University, Prague, Czech Republic; 4 Department of Biology, University of Rome Tor Vergata, Rome, Italy; 5 Department of Medicinal Chemistry, Faculty of Pharmacy, University of Athens, Greece; 6 Department of Chemistry, Sapienza University of Rome, Rome, Italy; 7 Institute of Biomolecular Chemistry, National Research Council (CNR), Rome, Italy; 8 Department of Physiology and Pharmacology “Vittorio Erspamer”, Sapienza University of Rome, Rome, Italy; Universidade Federal do Rio de Janeiro, Brazil

## Abstract

Natural polyphenol compounds are often good antioxidants, but they also cause damage to cells through more or less specific interactions with proteins. To distinguish antioxidant activity from cytotoxic effects we have tested four structurally related hydroxyflavones (baicalein, mosloflavone, negletein, and 5,6-dihydroxyflavone) at very low and physiologically relevant levels, using two different cell lines, L-6 myoblasts and THP-1 monocytes. Measurements using intracellular fluorescent probes and electron paramagnetic resonance spectroscopy in combination with cytotoxicity assays showed strong antioxidant activities for baicalein and 5,6-dihydroxyflavone at picomolar concentrations, while 10 nM partially protected monocytes against the strong oxidative stress induced by 200 µM cumene hydroperoxide. Wide range dose-dependence curves were introduced to characterize and distinguish the mechanism and targets of different flavone antioxidants, and identify cytotoxic effects which only became detectable at micromolar concentrations. Analysis of these dose-dependence curves made it possible to exclude a protein-mediated antioxidant response, as well as a mechanism based on the simple stoichiometric scavenging of radicals. The results demonstrate that these flavones do not act on the same radicals as the flavonol quercetin. Considering the normal concentrations of all the endogenous antioxidants in cells, the addition of picomolar or nanomolar levels of these flavones should not be expected to produce any detectable increase in the total cellular antioxidant capacity. The significant intracellular antioxidant activity observed with 1 pM baicalein means that it must be scavenging radicals that for some reason are not eliminated by the endogenous antioxidants. The strong antioxidant effects found suggest these flavones, as well as quercetin and similar polyphenolic antioxidants, at physiologically relevant concentrations act as redox mediators to enable endogenous antioxidants to reach and scavenge different pools of otherwise inaccessible radicals.

## Introduction

The radical scavenging properties of flavonoids and similar polyphenolic compounds are due to the presence of hydroxy groups on the aromatic ring system able to donate electrons or hydrogen atoms, and sometimes also to act as chelators of redox-active metal ions [1]–[5]. One class of flavonoids are the flavones which often are antioxidants, but flavones with methoxy groups also have other interesting biological effects. Despite the potential importance of this group of compounds relatively few studies on their intracellular antioxidant effects have been published; their use is limited by the scarce availability of most of them [6], [7].

Baicalein is a flavone derived from the roots of the plant *Scutellaria baicalensis*, one of the most popular traditional medicine herbs used in China. It has a very potent antioxidant activity compared to most other flavonoids, causing attenuation of oxidative stress effects in cardiomyocytes, and neuroprotection and anxiolytic effects in neuronal cells, and it is a mild relaxant compound in the neuromuscular system and skeletal muscles [8]–[10]. The dry root of *S. baicalensis* also contains other flavones (in particular baicalin, wogonin and wogonoside); it has been widely used in different pathological conditions such as hyperlipemia, atherosclerosis, hypertension, and inflammatory diseases [10]. Both baicalein and *S. baicalensis* extracts show cytostatic activity for many types of cancer cells in culture, and are able to inhibit proliferation of different human myeloma cell lines and other types of tumors *in vivo* [10]–[12]. Interestingly only little such inhibition was seen for non-tumor cells, even at high doses [13]; this low toxicity for non-tumor cells makes baicalein a very interesting antitumor drug candidate. The anti-tumor activity appears to be a multifactorial process involving several mechanisms, such as cell cycle inhibition, scavenging of superoxide, the inhibition of the redox-regulated transcription factor NF-κB, but also pro-oxidant activity causing induction of apoptosis [10].

Other natural flavones have been less characterized as to their antioxidant properties. However, it is known that such polyphenol compounds besides their protective effects due to radical scavenging also produce various side effects responsible for the cytotoxicity of many of them. How much of this toxicity depends on the high concentrations employed *in vitro*, and how important it may be *in vivo* is not clear. Their antiproliferative effect may be directly related to the general cytoxicity of these compounds, independently of their antioxidant properties; therefore their adverse effects must be examined before they are evaluated as therapeutic tools.

**Figure 1:**
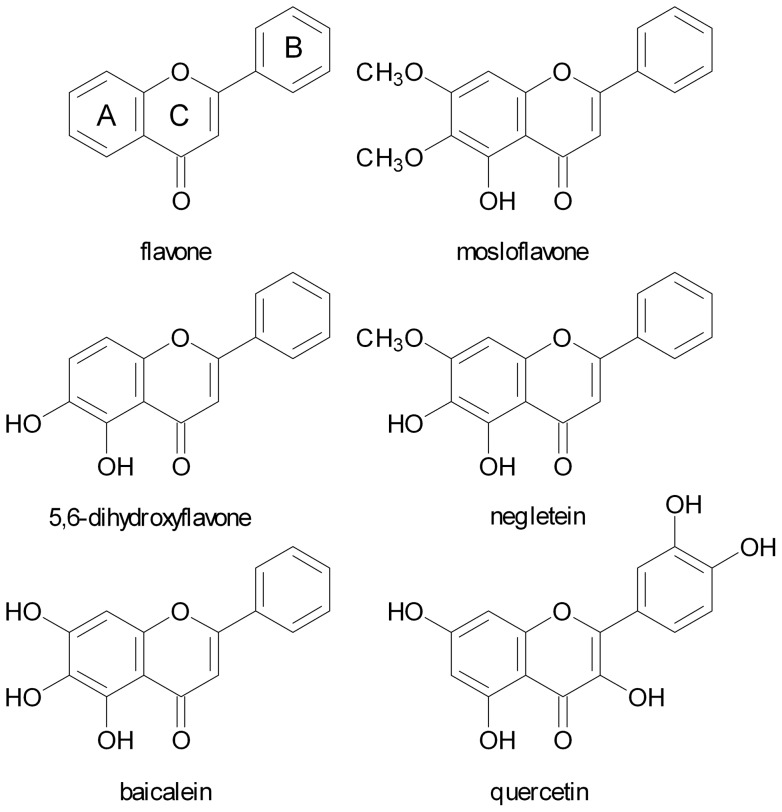
Antioxidant molecules. Structures of the flavone ring system, the four flavones tested, and the flavonol quercetin.

The potential importance of baicalein and similar molecules, together with the lack of information on the effects of flavones in general, prompted us to examine the antioxidant properties of four structurally related flavones: mosloflavone, negletein, 5,6-dihydroxyflavone, and baicalein. The synthesis of these compounds by a bromination/methoxylation procedure was recently published by some of the authors of the present paper [14]; the structures differ only by the presence of hydroxy or methoxy groups at the positions 5, 6, and 7 of the A ring (Fig. 1). In contrast to all previous studies we have used a wide range dose dependence approach, covering flavone concentrations from 1 pM to 10 µM, and thus including concentrations that correspond to the levels of flavonoids found in human plasma *in vivo* [15].

## Results

### Wide concentration range antioxidant activity evaluation

The effects of polyphenolic compounds on cells may depend very much on the type of cells studied, and it is therefore useful to test completely different cell types, in order to evaluate whether the results obtained are cell-specific or general features. We carried out experiments in parallel testing the antioxidant activity on L-6 myoblasts, a nontumor cell line from rat skeletal muscle, and on THP-1 monocytes, a human cell line of leukemic origin. The high catabolic activity level of muscle cells is accompanied by enhanced mitochondria-based production of reactive oxygen species (ROS), which makes the antioxidant defense an important factor. Monocytes, on the other hand, are directly exposed to the relatively higher levels of flavonoids that may be reached in the blood, and therefore the cytotoxic effects could be a more important issue for this type of cells.

The antioxidant activity of the flavones was measured using a standard intracellular fluorescent probe assay, in which the cells were exposed to a strong oxidative stress by addition of the radical generator cumene hydroperoxide. This gave rise to a rapid increase in intracellular fluorescence measured over a 10 min period, and the ability of the flavones to eliminate the increased ROS production was determined directly by their capacity to prevent this fluorescence increase (16).

**Figure 2:**
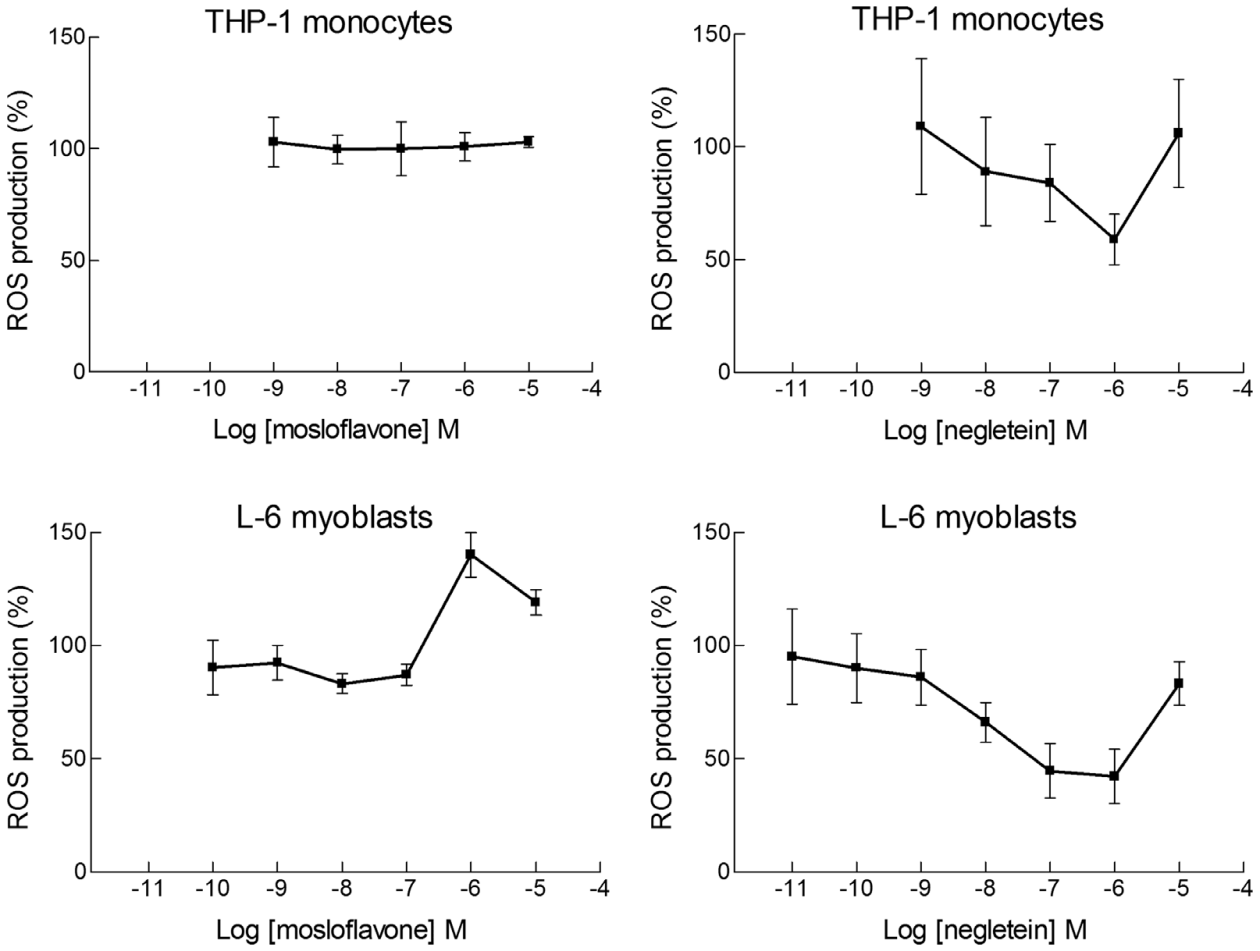
Dose-dependence curves for elimination of ROS by mosloflavone and negletein. Wide concentration range dose-responses of mosloflavone (**Left**) and negletein (**Right**) effects on ROS production measured with the dichlorodihydrofluorescein (DCF) method in L-6 myoblasts and THP-1 monocytes. Oxidative stress was induced by cumene hydroperoxide (200 µM); the change in fluorescence was measured for 10 min and reported as percentage with respect to the variation of fluorescence caused by cumene hydroperoxide in the absence of flavones. Data are reported as mean ± SD of 5–10 different experiments. For negletein *p*<0.05 at least, as from a Student's *t* test, from 10^−8^ M to 10^−6^ M for L-6 cells, and *p*<0.01 at 10^−6^ M for THP-1 cells.

Mosloflavone, which has a single hydroxy group and two methoxy groups in *ortho* position, did not show any intracellular antioxidant activity with either THP-1 or L-6 cells exposed to cumene hydroperoxide. Actually, in the myoblasts a pro-oxidant effect seemed to be caused by this compound in the micromolar range (Fig. 2, left panels). For negletein we found a limited dose-dependent antioxidant activity, reaching a maximum of about 50% at a concentration of 1 µM. The behavior was similar for L-6 and THP-1 cells, and for both cell types the effect was not observed at higher concentrations (Fig. 2, right panels).

**Figure 3:**
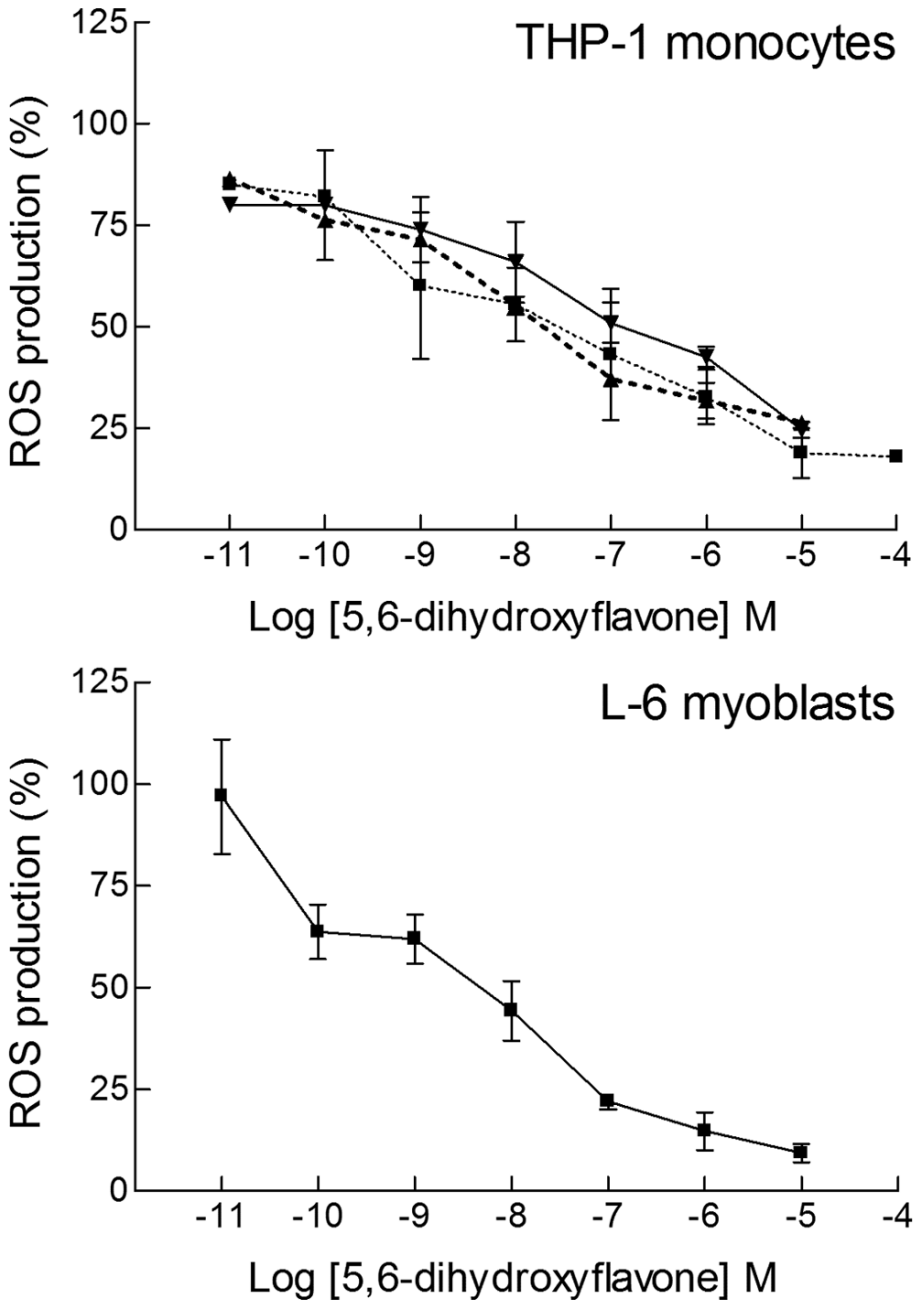
Dose-dependence curves for elimination of ROS by 5,6-dihydroxyflavone. Wide concentration range dose-responses of the antioxidant activity of 5,6-dihydroxyflavone in THP-1 monocytes (**Upper panel**) and in L-6 myoblasts (**Lower panel**). The oxidative stress was induced and measured as in Fig. 2. In addition to monocytes (▪) the upper panel also includes results obtained for differentiated macrophages after PMA treatment for 24 h (▴) or 72 h (▾). Data are reported as mean ± SD of 5–10 different experiments. *p*<0.05 at least, as from a Student's *t* test, starting from 10^−10^ M for both L-6 cells and THP-1 cells, at any stage of differentiation.

**Figure 4:**
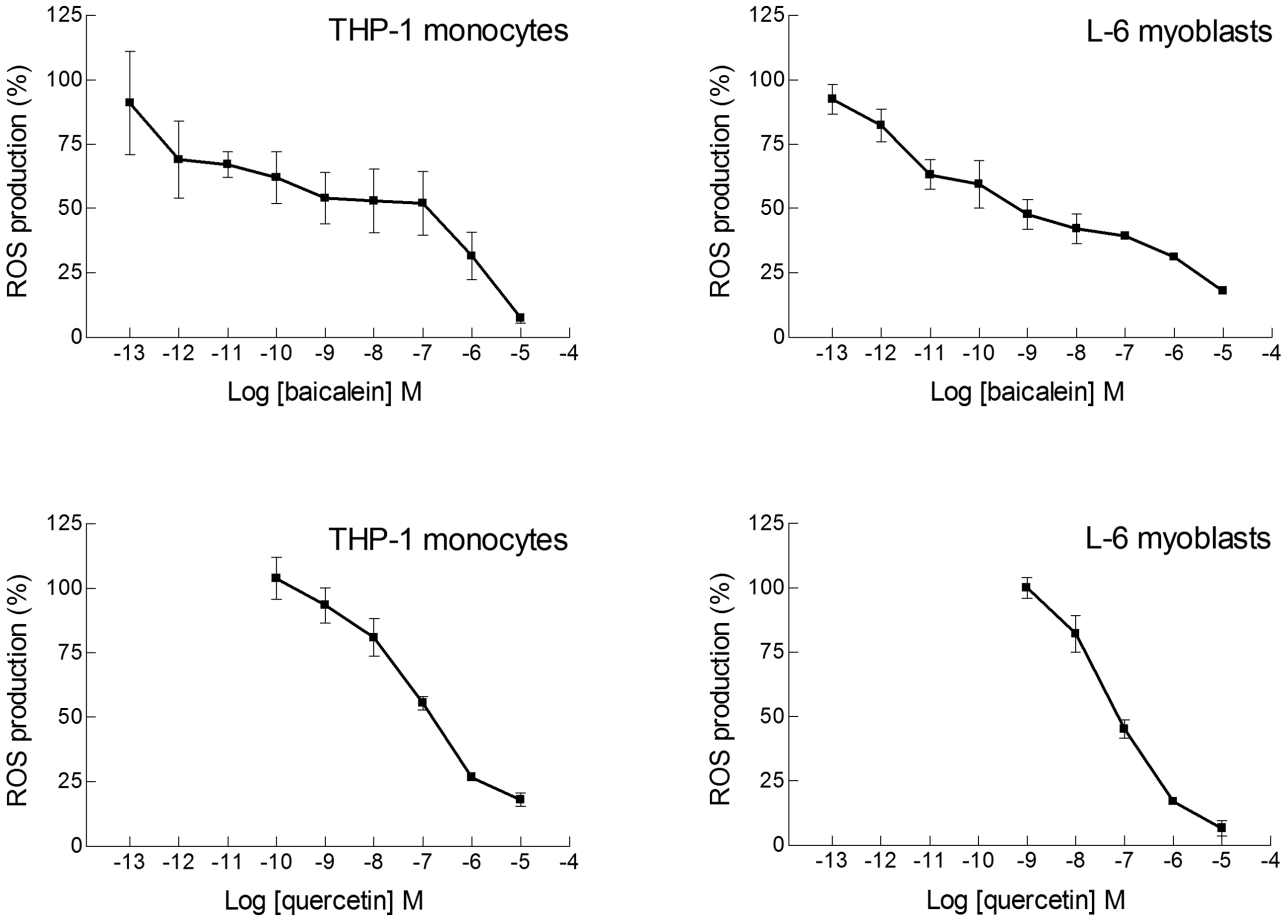
Dose-dependence curves for elimination of ROS by baicalein and quercetin. Wide concentration range dose-response curves for the antioxidant activity of baicalein (**Upper panels**) and quercetin (**Lower panels**) in THP-1 monocytes and L-6 myoblasts. The oxidative stress was induced and measured as in Fig. 2. Data are reported as mean ± SD of 5–10 different experiments. *p*<0.01 at least, as from a Student's *t* test, starting from 10^−12^ M for baicalein with both L-6 and THP-1 cells, and starting from 10^−8^ M for quercetin with both L-6 and THP-1 cells.

The 5,6-dihydroxyflavone has the catechol motif, two hydroxy groups in *ortho* position, which is considered one of the best features for an antioxidant compound, and very good antioxidant activity was found over a wide concentration range, significant even down to 100 pM with both cell types (Fig. 3). For this compound the activity was tested not only on undifferentiated monocytes but also on the differentiated cells, macrophages, obtained after treatment of THP-1 cells with phorbol 12-myristate 13-acetate (PMA) for 24 or 72 hours. The efficiency of 5,6-dihydroxyflavone was very good also in macrophages; only 10–20% of the oxidative stress produced by cumene hydroperoxide remained in differentiated and undifferentiated monocytes, as well as in myoblasts, after treatment with 10 µM 5,6-dihydroxyflavone (Fig. 3). For baicalein the situation was still more interesting. With three adjacent hydroxy groups baicalein was an excellent antioxidant in both L-6 and THP-1 cells, and in particular it was surprisingly efficient at extremely low concentrations. The antioxidant effect became significant already at 1–10 pM, with a decrease in DCF fluorescence of approximately 30–35% (Fig. 4). A comparison with the well-known flavonoid antioxidant quercetin showed that for both myoblasts and monocytes 50% reduction of the oxidative stress corresponded to 10^−7^ M quercetin, whereas for baicalein the same effect was obtained at 10^−9^ M (Fig. 4), suggesting that baicalein was a much better radical scavenger. However, at concentrations in the micromolar range quercetin was actually more efficient than baicalein in L-6 myoblasts; essentially the flavonol quercetin gave a much steeper dose-dependence curve than the flavones. For 5,6-dihydroxyflavone and baicalein we did not observe any loss of antioxidant effect at the highest concentrations tested (10 µM), in contrast to the results obtained for mosloflavone and negletein.

### Evaluation of cytotoxicity

**Figure 5:**
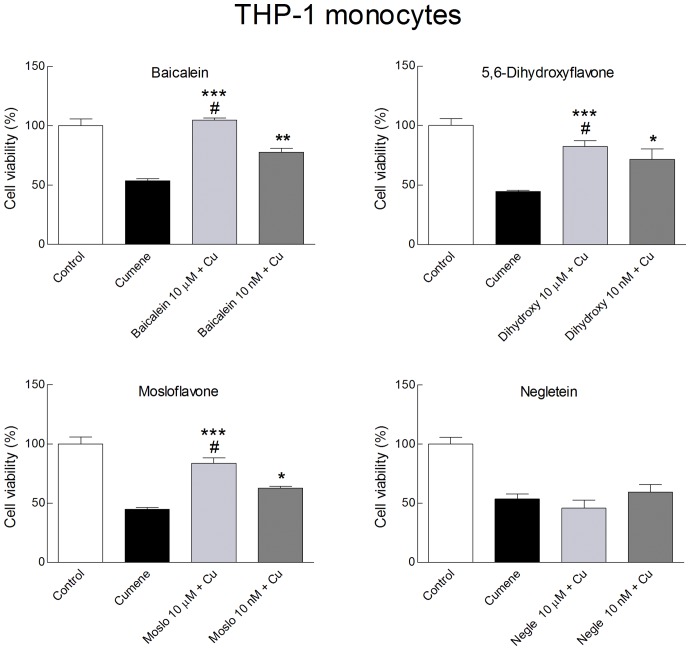
Cytotoxicity of flavones in monocytes. Effects of the four hydroxyflavones on the survival of THP-1 monocytes, as determined by the MTT assay. Cells were plated in 6-wells plates using 1×10^6^ cells/well and treated with the flavones (10 µM or 10 nM) for 30 min at 37° C, then cumene hydroperoxide was added at the final concentration of 200 µM for additional 30 min at 37°C. Then MTT solution (0.5 mg/ml final concentration) was added and incubation was carried out at 37°C for 3–4 h. Results represent cell viability and are given as percentage relative to untreated controls. Data are mean ± SD of 3 different experiments. The statistical significance was evaluated by a Student's *t* test; where not indicated the differences were not significant, except for the difference of Control *vs.* Cumene hydroperoxide (Cu) that was always significant. #not statistically different from Control; *p<0.05 with respect to cumene hydroperoxide alone; **p<0.01 with respect to cumene hydroperoxide alone, ***p<0.001 with respect to cumene hydroperoxide alone.

A toxicity test based on 3-(4,5-dimethylthiazol-2-yl)-2,5-diphenyltetrazolium bromide (MTT) gave information on the capability of the four compounds to protect the cells against oxidative stress, in terms of viability or proliferation. For two of the flavones the behavior in the MTT assays corresponded to the antioxidant activity, but interestingly the two others produced contrasting effects. In THP-1 monocytes exposed to cumene hydroperoxide both 5,6-dihydroxyflavone and baicalein were able to maintain cell viability completely at a concentration of 10 µM, and partial protection was seen even at concentrations as low as 10 nM (Fig. 5, upper panels). Surprisingly also mosloflavone at 10 µM provided full protection of these cells, and improved viability significantly (*p*<0.02) even at the lowest concentration tested (10 nM). In contrast negletein, at any concentration tested, was unable to protect the cells against the oxidative stress due to cumene hydroperoxide (Fig. 5, lower panels).

**Figure 6:**
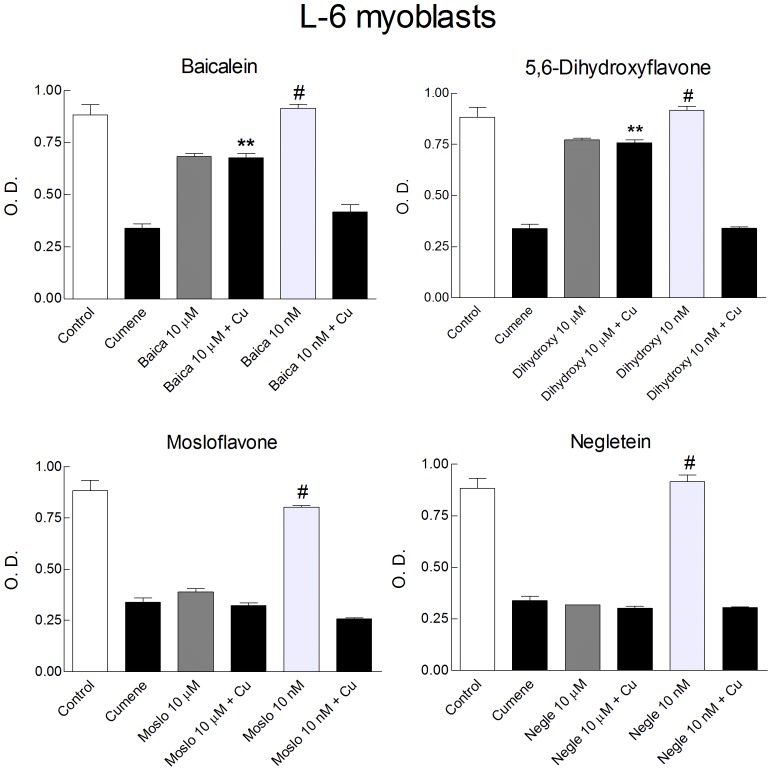
Cytotoxicity of flavones in myoblasts. Effects of the four hydroxyflavones on the survival of L-6 cells, as determined by the MTT assay. The concentration of cumene hydroperoxide was 27.5 µM, since preliminary experiments showed that higher concentrations were too toxic for these cells, and incubation with MTT was carried out for 24 h. Data are given as optical density (O.D.) rather than viability, because the values measured also include a component due to proliferation of the myoblasts. The bar graph shows data from a representative experiment with measurements carried out in triplicate. Data are mean ± SD of 3 different experiments. #not statistically different from Control; **p<0.01 with respect to cumene hydroperoxide alone.

Similar experiments were made for L-6 myoblasts, but because of the higher sensitivity of this cell line dose-response experiments had to be carried out to find a concentration of cumene hydroperoxide able to produce significant oxidative stress while preserving sufficient cell viability during the procedure of the MTT assay. Based on these experiments, not shown here for the sake of brevity, a cumene hydroperoxide concentration of 27.5 µM was chosen. Because of the longer incubation time used with myoblasts the results do not directly indicate viability, but may also include a component due to cell proliferation. In some aspects the behaviour of the antioxidants in myoblasts were similar to those found with THP-1 cells, but other results were unexpected. High levels (10 µM) of the good antioxidants baicalein and 5,6-dihydroxyflavone protected the cells against oxidative stress although complete protection was not observed, probably because the antioxidants alone gave rise to a decrease in cell viability/proliferation of 20–25% (Fig. 6, upper panels). Low concentrations of baicalein and 5,6-dihydroxyflavone (10 nM) did not cause such toxic effects, but at this level they were ineffective against the oxidative stress. At variance with the results found for THP-1 cells, mosloflavone did not protect L-6 cells against cumene hydroperoxide even at high concentrations; on the contrary both mosloflavone and negletein at 10 µM reduced cell viability/proliferation drastically (Fig. 6, lower panels). Interestingly, the cytotoxic effect of the flavones combined with the oxidative stress of cumene hydroperoxide did not appear to produce any cumulative effect in the L-6 myoblasts.

**Figure 7:**
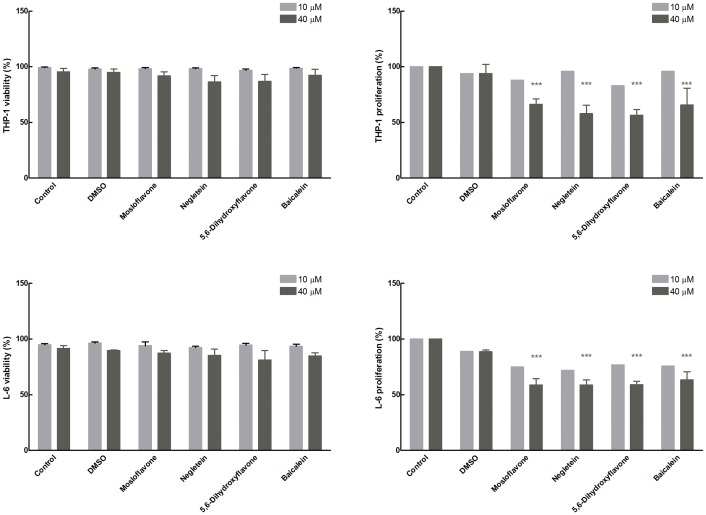
Direct cytotoxic effects of flavones. Direct effects of the four hydroxyflavones on THP-1 monocytes (**Upper panels**) and L-6 myoblasts (**Lower panels**) in the absence of any oxidative stress. Both cell viability (**Left panels**) and cell proliferation (**Right panels**) are shown as determined by the Trypan Blue assay; in these experiments cumene hydroperoxide was not added to the samples. The flavone concentration was 10 µM or 40 µM as indicated. Two types of control samples were included, one without any additions (control) and one with addition of a volume of the vehicle (DMSO) corresponding to the quantity added with the flavones. The data are shown as the mean ± SD of 3 different experiments with measurements carried out in triplicate, ***p<0.001 with respect to the corresponding control cells. For cell proliferation with 10 µM flavones a single representative experiment measured in triplicate is shown (statistical significance not calculated).

In addition to the MTT assay, cell viability and cell proliferation were determined for both cell lines also with the Trypan Blue exclusion test. This assay is based on a simple principle that Trypan Blue dye is excluded by viable cells but is taken up and retained by dead cells, which lack an intact membrane and thus appear blue. The direct toxic effects of the flavones on both THP-1 monocytes and L-6 myoblasts (Fig. 7) were evaluated in the absence of an oxidative stress (without addition of cumene hydroperoxide). At the two concentrations tested, 10 µM and 40 µM, none of the four hydroxyflavones affected cell viability (Fig. 7, left panels). However, cell proliferation was found to be significantly inhibited using 40 µM flavones (Fig. 7, right panels). Interestingly this decrease in proliferation was approximately the same (35–45%) for all the four flavones, indicating that this effect was not connected with their antioxidant properties.

**Figure 8:**
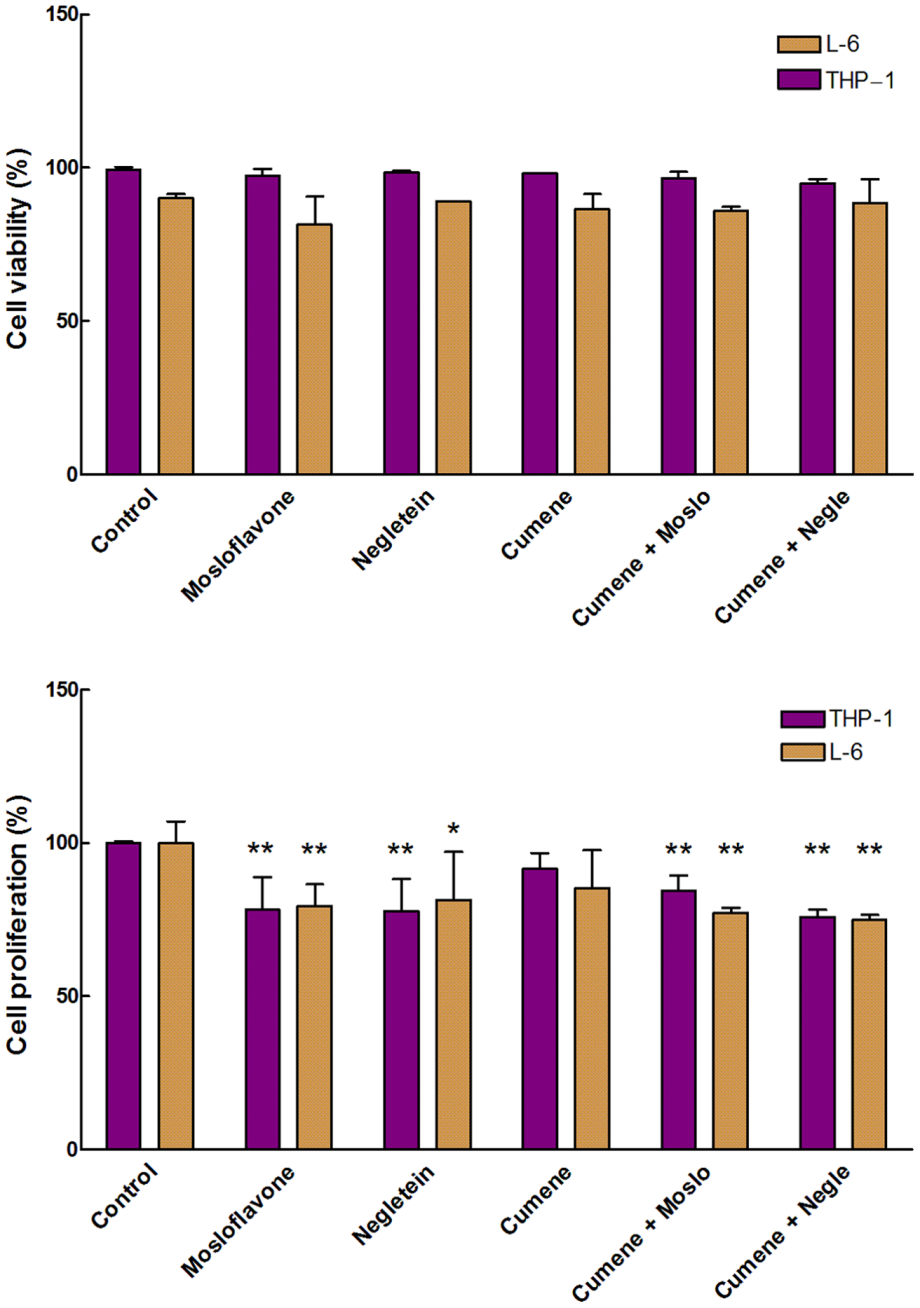
Cytotoxicity of flavones combined with cumene peroxide. Viability (**Upper panel**) and proliferation (**Lower panel**) of THP-1 monocytes and L-6 myoblasts treated with 10 µM of either mosloflavone or negletein with or without cumene hydroperoxide (27.5 µM), measured as described in Fig. 7. **p<0.01 with respect to the corresponding control; *p<0.05 with respect to the corresponding control.

The Trypan Blue assay was also used to compare the toxicity of cumene hydroperoxide in the two cell lines under identical conditions, using a low concentration of cumene hydroperoxide (27.5 µM) in combination with 10 µM of either mosloflavone or negletein, the two flavones without good intracellular antioxidant properties. Under these conditions there are no effects of cumene hydroperoxide and flavones on viability in the short time range (Fig. 8, upper panel) but a decrease in proliferation of approximately 20% is seen in samples containing flavones (Fig. 8, lower panel). Also in this case the addition of both cumene hydroperoxide and a flavone did not produce any cumulative effect.

**Figure 9:**
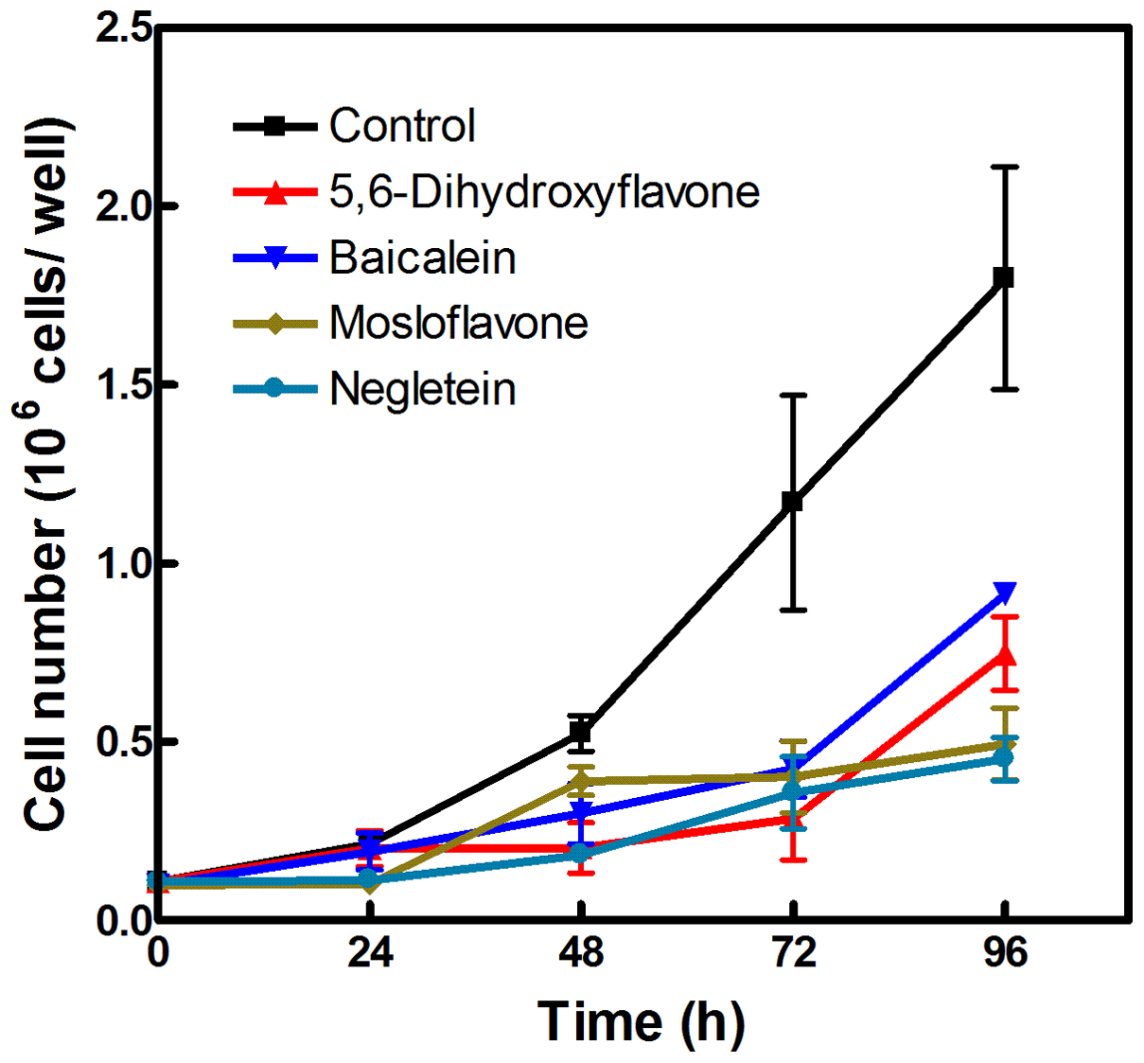
Long-term effects of flavones on proliferation. L-6 cells were grown for four days in the presence of 10 µM of either baicalein (▾), 5,6-dihydroxyflavone (▴), mosloflavone (♦) or negletein (•); an equivalent volume of DMSO was added to the control cells (▪).

The cytotoxic effect of flavones on proliferation became more prominent with time, and after 3 days the growth of flavone-treated cell was approximately 70% less compared to the controls (Fig. 9). Interestingly, cells treated with baicalein or 5,6-dihydroxyflavone apparently resumed normal growth after 3 days, while cells treated with mosloflavone or negletein continued a slow pace of proliferation; perhaps this effect is due to the generally slower metabolism of methoxylated compounds.

### Radical scavenging by flavones *in vitro*

**Figure 10:**
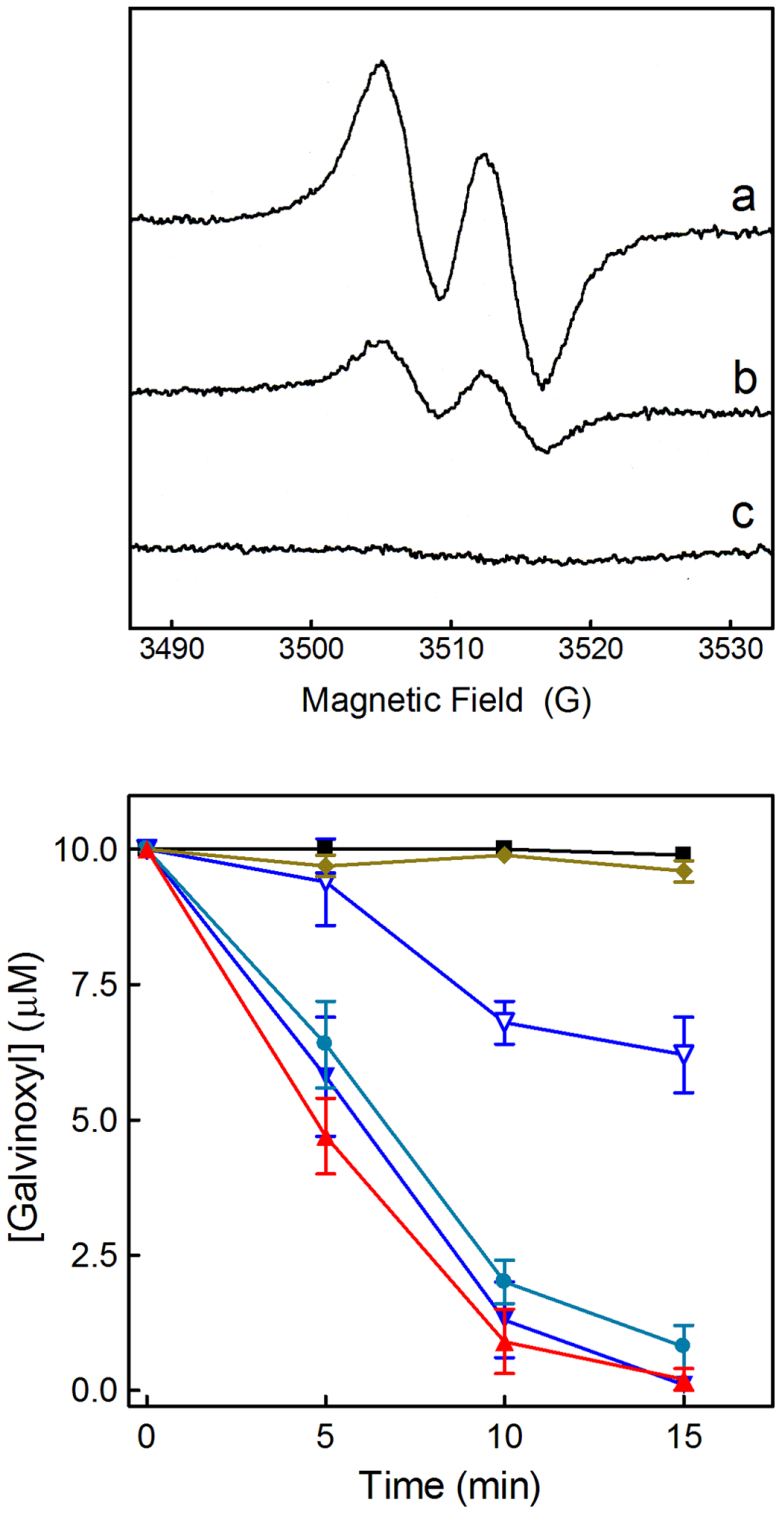
Antioxidant activity measured by EPR spectroscopy. **Upper panel:** EPR spectra showing the reaction between galvinoxyl and antioxidants after 5 min incubation. (**a**) 10 µM galvinoxyl in ethanol; (**b**) with the addition of 2 µM 5,6-dihydroxyflavone; (**c**) with the addition of 1.0 µM quercetin. For each spectrum 4 scans were accumulated [18]. The reduced product, galvinol, is not a radical, therefore it does not show an EPR spectrum. In theory it should be possible to see also the radical of the oxidized antioxidant, but almost all radicals of flavonoids and most other polyphenolic compounds are too reactive to be detected in this assay; their EPR spectra are not visible when the antioxidants are used in the micromolar range. The capacity of a compound to eliminate the galvinoxyl radical gives a relative measure of its general radical scavenging activity, although the intracellular antioxidant effect may depend on other factors and mechanisms. **Lower panel:** Kinetics of antioxidant activities of the four flavones measured by the EPR technique. The data show the concentration of galvinoxyl remaining in the samples at different times after addition of flavones, and are given as mean ± SD of 3 experiments. Control 10 µM galvinoxyl in ethanol (▪), and after addition of mosloflavone 10 µM (♦), negletein 1 µM (•), 5,6-dihydroxyflavone 1 µM (▴), baicalein 1 µM (▾), or baicalein 0.1 µM (▵). In samples containing 10 µM of either negletein, 5,6-dihydroxyflavone or baicalein the galvinoxyl signal disappeared completely within 30 s.

The ability of an antioxidant compound to act as a scavenger of radicals can be determined directly by a simple chemical assay, where the compound reacts with a relatively stable radical such as 2,2-diphenyl-1-picrylhydrazyl (DPPH) or galvinoxyl [16], [17]. The amount of radical eliminated can be measured precisely by EPR spectroscopy. Typical EPR spectra of galvinoxyl are shown in Fig. 10; the height of the signal is directly proportional to the concentration of the radical. In the presence of a suitable antioxidant compound galvinoxyl is reduced and the galvinoxyl spectrum decreases or disappears completely, depending on the reducing power of the antioxidant, the quantity added and the reaction time.

The flavones were tested in the concentration range 0.1–10 µM against 10 µM galvinoxyl, and the galvinoxyl radical remaining in the samples was measured after 5, 10, and 15 min (Fig. 10). Baicalein, 5,6-dihydroxyflavone, and negletein were all very efficient, with almost immediate elimination of the radical at the highest concentration tested, and very little galvinoxyl remaining after 15 min incubation with 1.0 µM of these three flavones. Baicalein even showed considerable scavenging activity at a concentration of 0.1 µM. Only mosloflavone did not work; this was expected as its single hydroxy group would not be predicted to be particularly reducing. Similar results were obtained for the reaction of the four flavones with DPPH (data not shown). For baicalein and 5,6-dihydroxyflavone the EPR result were in good agreement with those obtained with the intracellular DCF measurements. Negletein, however, was far more efficient as radical scavenger here than in the cells.

**Figure 11:**
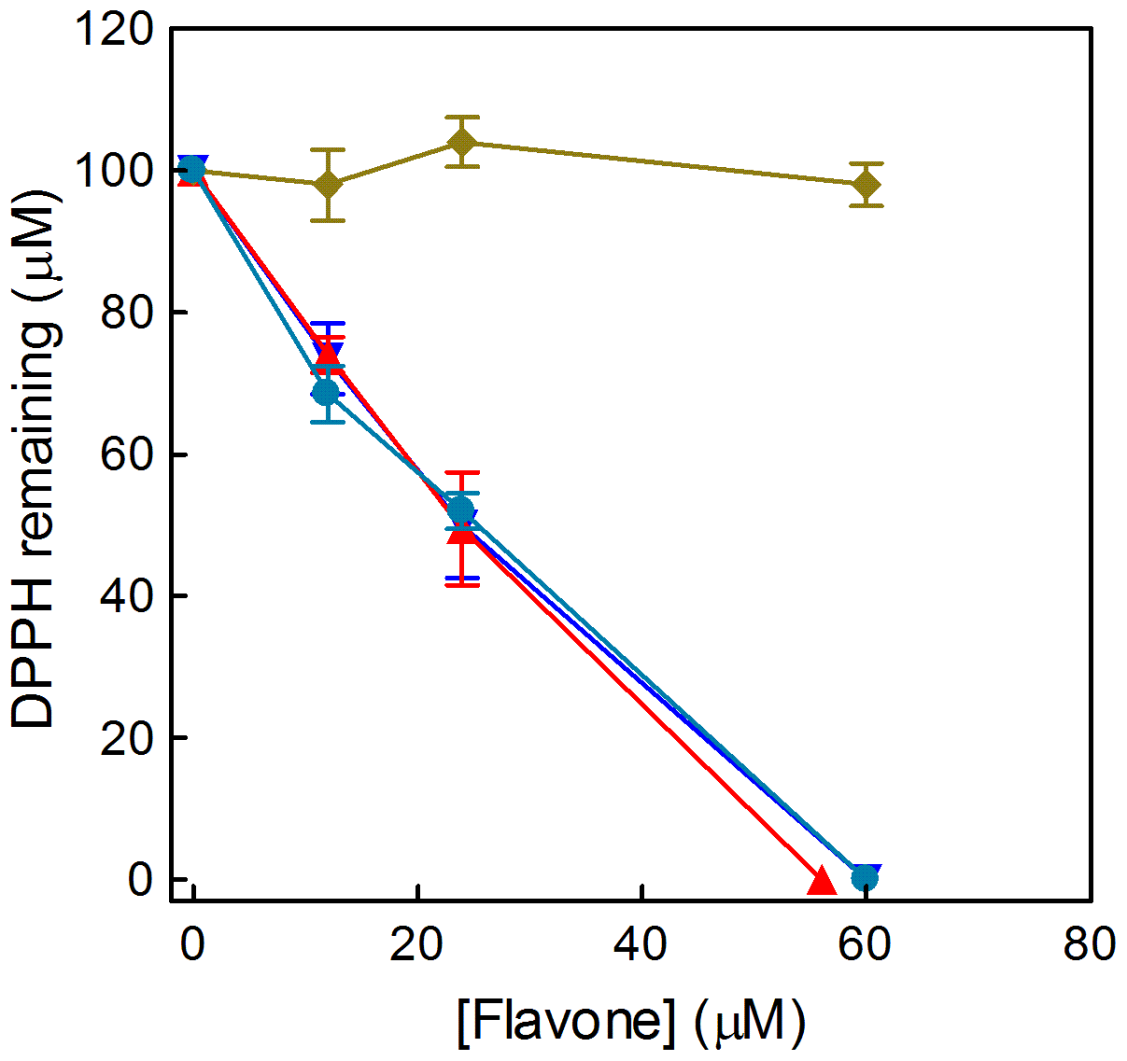
Antioxidant activity measured by the DPPH assay. The dose dependent radical scavenging activity of the four flavones was measured by the decrease in absorption at 517 nm. The data show the concentration of DPPH remaining in the samples 20 min after addition of mosloflavone (♦), negletein (•), 5,6-dihydroxyflavone (▴) or baicalein (▾). In control samples containing only 100 µM DPPH in 95% ethanol no change in the spectrum was detected within the time of the measurements.

As an alternative to the EPR experiments, the radical-scavenging efficiency of the four flavones was also determined using a standard spectrophotometric method based on DPPH reduction and using higher concentrations of flavones (Fig. 11). The results confirm the lack of activity of mosloflavone and the strong antioxidant activities of baicalein, 5,6-dihydroxyflavone and negletein; again the efficiency of negletein in this chemical assay contrasts with the relatively bland effect observed for both cell types tested (Fig. 2).

## Discussion

A surprising finding of the present study is the strong intracellular antioxidant effect at extremely low levels of baicalein and 5,6-dihydroxyflavone. With both cell types studied a concentration of 100 pM baicalein produced a decrease in intracellular DCF fluorescence of approximately 40% whereas 5,6-dihydroxyflavone at the same concentration caused around 25% less DCF fluorescence. For baicalein a significant effect was observed even at a concentration of 1 pM; to our knowledge antioxidant effects at this level have never been demonstrated before, in any type of chemical or biological systems.

The antioxidant activity was determined here with well-established standard methods, but as a new approach the intracellular effect was determined over an extremely wide concentration range, spanning 8 orders of magnitude. The 5,6,7-trihydroxyflavone baicalein showed outstanding antioxidant activity with all methods, but 5,6-dihydroxyflavone was only slightly less efficient. In theory three adjacent hydroxyls (galloyl or pyrogallol motif) should confer a better radical scavenging ability than two adjacent hydroxyls (catechol), but in practice this is not always seen for flavonoids; quercetin with a 3′,4′-dihydroxy-substituted B ring is often found to be a stronger antioxidant than myricetin, which has a 3′,4′,5′-trihydroxy-substituted B ring [18]. Negletein has the same catechol motif as 5,6-dihydroxyflavone, and the two compounds gave almost identical results with EPR and spectrophotometric assays, but negletein was much less efficient in the cells. DCF fluorescence in both monocytes and myoblasts actually increased at concentrations above 1 µM negletein, indicating a pro-oxidant effect with increased reactive oxygen species (ROS) production. Mosloflavone with one single hydroxy group never showed antioxidant activity in any of these assays.

The protection provided by baicalein and 5,6-dihydroxyflavone against oxidative stress in THP-1 monocytes was in agreement with their antioxidant effects. Partial protection was still observed even at 10 nM of these flavones, consistent with the approximately 50% decrease in DCF fluorescence observed at this concentration. More surprisingly mosloflavone protected the viability of THP-1 cells with the same efficiency as 5,6-dihydroxyflavone, in spite of its apparent lack of antioxidant activity. The explanation could be that mosloflavone becomes metabolized to baicalein in THP-1 cells during the MTT assay incubation period. Several cytochrome P450 isoforms are able to demethylate methoxylated flavones, and partially methoxylated flavones like mosloflavone are typically metabolized rapidly [19]. This effect obviously depends on the cytochrome P450 profile of the cell; we did not observe any protection by mosloflavone in L-6 myoblasts, and negletein did not protect any of the cell types, in spite of its activity in the chemical assays. On the contrary, both mosloflavone and negletein at a concentration of 10 µM were cytotoxic in L-6 cells, and also baicalein and 5,6-dihydroxyflavone caused a small but significant decrease in cell viability/proliferation, although they were still able to suppress the additional toxicity caused by cumene hydroperoxide (Fig. 6). We conclude that antioxidant and cytotoxic effects of these flavones depend very much on the cell model chosen. Baicalein has not been tested before with L-6 myoblasts or muscle tissue, whereas the only published study with THP-1 cells showed that 200 µM baicalein induced cell cycle arrest at G2/M phase and apoptosis [20]. Nothing is known of the biological activities of the three other flavones used in the present work, except that negletein has a potent inhibitory effect (IC_50_  = 3.9 µM) on the NFAT transcription factor [21].

The antioxidant activity of baicalein has been documented in many different cell models and *in vivo* [10], [22]–[24], although pro-oxidant effects have also been observed [10], [25]. Most studies on this flavone, however, have dealt with the effects on signal transduction pathways and regulatory enzymes involved in cell proliferation, differentiation and apoptosis, and baicalein has been found to downregulate or inhibit some key proteins including NF-κB, Bcl-2, pAkt, p38, JNK, ERK, inducible NO synthase and matrix metalloproteinases, whereas others like p53 and Bax showed increased expression [10], [26], [27]. But contrasting results such as decreased Bax/Bcl-2 ratios have recently been reported [28]; this confirms that baicalein effects should not be generalized on the basis of results obtained within a specific cell context. It is difficult to separate the antioxidant activity of baicalein from its effect on signal transduction pathways, in particular because of the high concentrations (25–300 µM) typically used in these studies. Only a few cases of direct interaction of baicalein with proteins have been discovered so far; it has been used for many years to inhibit 12-lipoxygenase activity in cells [29], and is a strong inhibitor of xanthine oxidase (IC_50_  = 3.1 µM) [30].

How can baicalein act as an antioxidant at a concentration of 1 pM? Baicalein is a strong iron chelator under physiological conditions and may be able to modulate iron homeostasis in the body and prevent Fenton-type reactions [31]–[33]. However, formation of baicalein-Fe complexes would require stoichiometric amounts of baicalein to sequester the micromolar levels of spurious or chelatable iron(II) in cells [18], and therefore cannot explain the protection seen at nanomolar concentrations. The wide range dose-dependence approach used here provides new information on the antioxidant mechanism. For both baicalein and 5,6-dihydroxyflavone the slope of the curves is remarkably flat (Figs. 3 and 4); increasing the concentration 100 times does not improve the antioxidant activity very much. If the antioxidant activity were due to activation or inhibition of a protein the affinity would have to be extremely high to induce effects at picomolar levels, essentially like a receptor. But then a 100 times higher concentration should produce drastic effects in terms of activation/inhibition, and this is not compatible with the low slope of the curves; furthermore the curves do not reach saturation even after increasing the concentration 10^6^ times. Differentiation of THP-1 monocytes to macrophages involves profound intracellular changes in signal transduction activities, but the wide range dose-dependence curves for the antioxidant activity are only minimally affected by this process. Taken together our data seem to exclude that the effects of baicalein and 5,6-dihydroxyflavone involve a protein-mediated response.

Other types of antioxidants have recently been shown to work at low nanomolar levels, such as tocotrienols [34] and bisarylamines [35]; however, those compounds have very low solubility in water and can be expected to accumulate in the membranes where their local concentration will be much higher. Novel water-soluble antioxidants developed by Skulachev and coworkers are also active at nanomolar concentrations, but these compounds were actually designed to accumulate in mitochondria [36]. At present there is no evidence for baicalein accumulation anywhere in the cells, and no reason to believe this might happen. Still, it is obvious that picomolar quantities of an antioxidant cannot reduce directly micromolar quantities of radicals. Considering that the intracellular antioxidants include micromolar levels of catalase, superoxide dismutase, glutathione peroxidases, urate, ascorbate and tocopherols, and millimolar levels of glutathione, it is also obvious that the addition of 0.000001 µM baicalein should not make any detectable contribution to the total antioxidant capacity. Clearly this flavone is somehow able to eliminate a part of the oxidative stress in cells, which the endogenous antioxidant defence system cannot cope with. We suggest that baicalein and 5,6-dihydroxyflavone may act as redox mediators that scavenge pools of radicals not accessible to endogenous antioxidants; the oxidized flavones can then be reduced back to the active form by intracellular reductants. This behaviour would be consistent with high activity at substoichiometric levels, and with the slopes of the dose-dependence curves.

It is likely that other natural antioxidants may work in the same way at low concentrations. In both myoblasts and monocytes the flavonol quercetin gives a different and much steeper wide range dose-dependence curve (Fig. 4), it has no activity below 10 nM but is more efficient than baicalein at micromolar levels. A simple chemical reaction mechanism cannot account for this result; it would require that the reaction constant(s) of baicalein should become smaller with increasing concentration. The different dose-dependence curves suggest that these two antioxidants target different intracellular radical pools. This difference in reactivity may reflect their structural differences; the reducing catechol moiety of quercetin is on the opposite side of the molecule compared to baicalein. A comparison of the four flavones studied reveals that the presence of two adjacent hydroxy groups in positions 5 and 6 on the A ring is a necessary but not sufficient requirement for intracellular antioxidant activity. Negletein shows the same chemical reactivity as 5,6-dihydroxyflavone towards the galvinoxyl radical, but the methoxy group in 7-position prevents negletein from reacting efficiently with the radicals that 5,6-dihydroxyflavone scavenges in the cells. The application of wide range dose-dependence curves can be used to characterize and distinguish the mechanism and targets of many different antioxidant types, and we believe that this tool may make it possible to establish their role as redox mediators, as well as the existence of different intracellular radical pools.

## Materials and Methods

### Materials

Mosloflavone (6,7-dimethoxy-5-hydroxyflavone), negletein (5,6-dihydroxy-7-methoxyflavone), 5,6-dihydroxyflavone (no trivial name) and baicalein (5,6,7-trihydroxyflavone) were synthesized through convergent synthesis starting from chrysin as previously reported [14]. Galvinoxyl, 2,2-diphenyl-1-picrylhydrazyl (DPPH), cumene hydroperoxide, quercetin, 3-(4,5-dimethylthiazol-2-yl)-2,5-diphenyltetrazolium bromide (MTT) and phorbol 12-myristate 13-acetate (PMA) were purchased from Sigma-Aldrich (St. Louis, MO). 2′,7′-Dichlorodihydrofluorescein diacetate (DCFH_2_-DA) was from Molecular Probes (Eugene, OR). Dulbecco's modified Eagle's medium, RPMI medium, antibiotics, and sterile plasticware for cell culture were from Flow Laboratory (Irvine, UK). Fetal Bovine Serum was from GIBCO (Grand Island, NY, USA).

### Cells in culture

L-6 myoblasts were obtained from American Type Culture Collection (Rockville, MD, USA). Cells were seeded in 75 cm^2^ tissue culture flasks and grown in Dulbecco's modified Eagle's medium supplemented with 10% fetal bovine serum, 100 µg/ml streptomycin and 100 U/ml penicillin, in humidified atmosphere with 5% CO_2_ at 37°C. Cells reached confluency after 5 days (approximately 6×10^6^ cells/flask) and were kept in culture as myoblasts by continuous passages at preconfluent stages [37].

THP-1 cells, originally bought from American Type Culture Collection, were a kind gift from Dr. Giorgio Mangino, Laboratory of Virology, Department of Biology, University Roma Tre, and grown in suspension in RPMI-1640 medium with 10% FBS, 100 µg/ml streptomycin and 100 U/ml penicillin, in humidified atmosphere with 5% CO_2_ at 37°C. For experiments with differentiated macrophages the cells were grown for 24 or 72 h in the presence of 0.1 µM PMA [38]. The THP-1 monocytes were passaged twice a week by 1∶4 dilutions and reseeded; cells from passages 7–23 were used for the experiments.

### Intracellular ROS determination

The method used was a standard assay based on the intracellular fluorescent probe DCF [39]–[42]. For L6 myoblasts the medium was discarded and cells were washed twice with 5 ml phosphate buffered saline (PBS) containing 5.0 mM glucose (PBS-glucose), at 37°C. Cells were gently scraped off with PBS-glucose at 37°C and centrifuged at 1200 rpm for 5 min (about 100× *g*), the supernatant was discarded and the pellet resuspended in PBS-glucose. Incubation with the probe DCFH_2_-DA at a final concentration of 10 µM (from a stock solution of 10 mM in dimethyl sulfoxide) was carried out for 30 min in the dark at 37°C, as previously reported [16]. At the end of the incubation cells were washed twice, centrifuged at 1200 rpm for 5 min, and the final cell pellet was resuspended in PBS-glucose. Before the experiments cells recovered at room temperature for 45 min in the dark.

The THP-1 monocytes were centrifuged at 1200 rpm for 10 min; the supernatant was discarded and cells were washed twice with 5 ml PBS-glucose at 37°C to remove the serum, that might affect the action of the fluorescent probe. After the last centrifugation the pellet was resuspended in PBS-glucose and the probe DCFH_2_-DA was added at 10 µM final concentration; from that point onwards the protocols for the two cell types were identical.

Intracellular fluorescence was measured with a LS 50B luminescence spectrometer (Perkin–Elmer, Norwalk, CT). Excitation and emission wavelengths were set at 498 nm and 530 nm respectively, using 5 and 10 nm slits for the two light paths. The assay was carried out in 3 ml PBS-glucose containing 6×10^5^ cells with gentle magnetic stirring at 37°C. Cumene hydroperoxide diluted in dimethyl sulfoxide was used as radical generator (final concentration 200 µM); dimethyl sulfoxide at the concentrations used did not affect the fluorescence signal. Antioxidant activity of the flavonoids was determined by the decrease in intracellular DCF fluorescence, measured as ΔF/10 min, and calculated relative to the fluorescence change induced by 200 µM cumene hydroperoxide alone (100%). None of the compounds tested gave rise to fluorescence on their own. Before addition of cumene hydroperoxide, cells were pre-incubated with flavones at 37°C for 10 min.

### MTT assay

Cell viability was assessed for THP-1 monocytes and L-6 myoblasts by the MTT assay as previously reported [43]; the procedure was slightly different for the two cell lines due to the higher sensitivity of the L-6 cells to cumene hydroperoxide. L-6 cells were seeded in 96-wells plates at 10,000 cells/well in 200 µl growth medium containing 10% serum. After 24 or 48 h the medium was discarded, 200 µl PBS-glucose was added, and the cells were exposed to cumene hydroperoxide, with or without flavones at different concentrations. Results are reported as the mean ± SD of 3 experiments carried out in triplicate for the THP-1 cells, whereas a single experiment in triplicate is reported for the L-6 myoblasts.

### Trypan Blue and cell proliferation assay

The cells were seeded in 24-well plates in 1 ml of the growth medium. After 24 h, the cells were exposed to flavones at different concentrations (10 or 40 µM) and incubated for additional 24 h. Finally, the cell counts were made by the use of a Neubauer chamber and 0.4% Trypan Blue solution (Sigma). The cell viability was expressed as number of viable cells (unstained)/total cell number (stained and unstained) and indicated as percentage viability. Cell proliferation was expressed as percentage total number of cells found compared to the control group (100% untreated cells). Results are given as mean ± SD of 3 experiments for the 40 µM concentration, whereas a single experiment in triplicate is shown for the 10 µM concentration.

### Long-term proliferation assay

L-6 cells were seeded in 60×15 mm wells at a density of about 10^5^ cells/well. The following day the cells were exposed to flavones at a final concentration of 10 µM, and counted in a Neubauer chamber every 24 hours after mild trypsinization. The results are given as the mean ± SD of 3 different experiments carried out in duplicate.

### EPR spectroscopy

Systematic screening of all flavones was made using a final concentration of 10 µM in 95% ethanol in the presence of 10 µM galvinoxyl; efficient radical scavenging flavones were also tested at 1.0 or 0.1 µM. For some experiments DPPH (50 µM) was used as an alternative to galvinoxyl. Samples containing 50 µl solutions were drawn into glass capillaries, sealed, and measured using an ESP300 instrument (Bruker Spectrospin, Karlsruhe, Germany) equipped with a high sensitivity TM_110_ X-band cavity. EPR spectra were recorded at room temperature, using 0.6 G modulation, 1 mW microwave power, and a scan time of 42 s for a 30 G spectrum. Normally four spectra were accumulated for each measurement in order to obtain a suitable signal to noise ratio [16].

### DPPH reduction

Radical-scavenging activity of the four flavones was measured with a Beckman DU-800 spectrophotometer through the bleaching of DPPH at 517 nm [44]. Samples containing 100 µM DPPH in 95% ethanol and different concentrations (0–60 µM) of the flavone were incubated in the dark at room temperature for 10 min before measurements.

### Statistical analysis

All assays were carried out at least in triplicate and data were expressed as mean ± standard deviation (SD). Statistical significance was determined with a Student's *t* test using the GraphPad Prism 4.0 program.
